# Altered splicing machinery in lung carcinoids unveils NOVA1, PRPF8 and SRSF10 as novel candidates to understand tumor biology and expand biomarker discovery

**DOI:** 10.1186/s12967-023-04754-8

**Published:** 2023-12-04

**Authors:** Ricardo Blázquez-Encinas, Víctor García-Vioque, Teresa Caro-Cuenca, María Trinidad Moreno-Montilla, Federica Mangili, Emilia Alors-Pérez, Sebastian Ventura, Aura D. Herrera-Martínez, Paula Moreno-Casado, Marco A. Calzado, Ángel Salvatierra, María A. Gálvez-Moreno, Lynnette Fernandez-Cuesta, Matthieu Foll, Raúl M. Luque, Nicolas Alcala, Sergio Pedraza-Arevalo, Alejandro Ibáñez-Costa, Justo P. Castaño

**Affiliations:** 1 Maimónides Biomedical Research Institute of Córdoba (IMIBIC), Córdoba, Spain; 2 Department of Cell Biology, Physiology, and Immunology, University of Córdoba, Córdoba, Spain; 3 Reina Sofia University Hospital, Córdoba, Spain; 4 Pathology Service, Reina Sofía University Hospital, Córdoba, Spain; 5 Department of Clinical Sciences and Community Health, University of Milan, Milan, Italy; 6 Department of Computer Sciences, University of Córdoba, Córdoba, Spain; 7 Endocrinology and Nutrition Service, Reina Sofia University Hospital, Córdoba, Spain; 8 Thoracic Surgery and Lung Transplantation Unit, Reina Sofa University Hospital, Córdoba, Spain; 9 Rare Cancers Genomics Team (RCG), Genomic Epidemiology Branch (GEM), International Agency for Research On Cancer (IARC/WHO), Lyon, France; 10 CIBER Fisiopatología de La Obesidad y Nutrición (CIBERobn), Córdoba, Spain

**Keywords:** Neuroendocrine neoplasms, Pulmonary carcinoids, RNA splicing, NOVA1, PRPF8, SRSF10

## Abstract

**Background:**

Lung neuroendocrine neoplasms (LungNENs) comprise a heterogeneous group of tumors ranging from indolent lesions with good prognosis to highly aggressive cancers. Carcinoids are the rarest LungNENs, display low to intermediate malignancy and may be surgically managed, but show resistance to radiotherapy/chemotherapy in case of metastasis. Molecular profiling is providing new information to understand lung carcinoids, but its clinical value is still limited. Altered alternative splicing is emerging as a novel cancer hallmark unveiling a highly informative layer.

**Methods:**

We primarily examined the status of the splicing machinery in lung carcinoids, by assessing the expression profile of the core spliceosome components and selected splicing factors in a cohort of 25 carcinoids using a microfluidic array. Results were validated in an external set of 51 samples. Dysregulation of splicing variants was further explored in silico in a separate set of 18 atypical carcinoids. Selected altered factors were tested by immunohistochemistry, their associations with clinical features were assessed and their putative functional roles were evaluated in vitro in two lung carcinoid-derived cell lines.

**Results:**

The expression profile of the splicing machinery was profoundly dysregulated. Clustering and classification analyses highlighted five splicing factors: *NOVA1*, *SRSF1*, *SRSF10*, *SRSF9* and *PRPF8*. Anatomopathological analysis showed protein differences in the presence of NOVA1, PRPF8 and SRSF10 in tumor versus non-tumor tissue. Expression levels of each of these factors were differentially related to distinct number and profiles of splicing events, and were associated to both common and disparate functional pathways. Accordingly, modulating the expression of NOVA1, PRPF8 and SRSF10 in vitro predictably influenced cell proliferation and colony formation, supporting their functional relevance and potential as actionable targets.

**Conclusions:**

These results provide primary evidence for dysregulation of the splicing machinery in lung carcinoids and suggest a plausible functional role and therapeutic targetability of NOVA1, PRPF8 and SRSF10.

**Supplementary Information:**

The online version contains supplementary material available at 10.1186/s12967-023-04754-8.

## Background

Lung neuroendocrine neoplasms (LungNENs) comprise a heterogeneous group of tumors classified into four distinct types, according to their histological grade, by the 2021 WHO classification [1]: the well differentiated typical (G1) and atypical (G2) carcinoids, and the poorly differentiated large cell neuroendocrine carcinoma (LCNEC) and small cell lung cancer (SCLC) (both G3). Typical carcinoids are slow proliferating neoplasms that rarely spread beyond the lungs, whereas atypical carcinoids are more aggressive with higher rates of metastasis. Although both carcinoids are morphologically well differentiated, they are characterized by a distinct molecular signature, especially compared to poorly differentiated NENs. In particular, carcinoids have lower mutational burden than poorly differentiated neoplasms, but mutations in *MEN1* linked to loss of expression are relatively frequent (11–22%) [2]. Likewise, other chromatin remodeling genes are frequently mutated in typical (40%) and atypical carcinoids (22.2%), especially genes of the SWI/SNF complex and covalent histone modifiers [3, 4]. Gene expression analyses have also unveiled some molecular pathways altered in carcinoids, including mitotic spindle checkpoint or chromosomal passenger complex [5]. Some individual genes such as *CD44* and *OTP* have been shown to be downregulated in carcinoids, and their loss of expression, both at RNA and protein levels, are associated with poorer prognosis [6, 7]. The increasing information attained through genomic and transcriptomic approaches is providing a more precise picture of LungNENs, which may enable to refine and improve their classification, and could offer prognostic and predictive information [8–10]. However, the actual translational value of these discoveries is still limited and, therefore, novel avenues should be explored to better understand and combat these tumors [11–13].

In this scenario, the splicing of RNA and its related mechanisms are emerging as a novel and informative layer to enhance our molecular comprehension of cancer. In fact, RNAs require a maturation process that in more than 95% of genes includes alternative splicing, a complex and dynamic multistep sequential mechanism carried out and controlled by a macromolecular ribonucleoproteic machinery, the spliceosome, and hundreds of splicing factors, which enable the genesis of distinct variants from the same gene, thus increasing transcript and protein variety [14]. This dynamic stepwise process involves concerted actions by ribonucleoproteins and splicing factors to ensure a precise selection of intron–exon sequences and their subsequent enzymatic processing [14]. Specifically, 98% of introns are processed by the major spliceosome, while the remaining are spliced by the minor spliceosome, which share most of their components but differ in a limited set of U RNAs and accompanying splicing factors [15]. Interestingly, there is now ample evidence that alternative splicing is commonly dysregulated in all tumors and cancers examined [16, 17], including pancreatic NENs and SCLC [18–21]. This dysregulation may lead to the appearance of aberrant splicing variants imparting malignant properties to cancer cells, and has emerged as a transversal hallmark pervading all the other cancer hallmarks [20, 22–25]. To date, however, the possible dysregulation of alternative splicing, particularly its driving machinery, and its putative functional consequences in well differentiated pulmonary carcinoids remain unknown. In this study, we interrogated the status of the splicing machinery in pulmonary carcinoids and assessed the clinical associations and functional roles of a set of factors found to be altered, to test their potential as new biomarkers and therapeutic targets.

## Methods

### Patients and samples

A cohort of 25 human pulmonary carcinoids (11 typical, 8 atypical and 6 that could not be determined) was analyzed in this study (Discovery cohort). Samples were collected after surgery from 2005 to 2015 in the Reina Sofia University Hospital (Córdoba, Spain) and were immediately fixed with formaldehyde 10% solution and embedded in paraffin. Identification of tumor and non-tumor adjacent tissue as well as immunohistochemistry (IHC) and its assessment in these samples were performed by three different expert lung pathologists, following WHO criteria of 2021. This study was approved by the Ethics Committee of the Reina Sofia University Hospital and the Declaration of Helsinki guidelines were followed. Informed consent documentation was obtained from each of the patients involved in the study. Gene expression data from 51 human samples (including 31 typical and 11 atypical carcinoids, and 9 adjacent normal lung tissue), which served as a Validation cohort, were downloaded from Gene Expression Omnibus (GEO) under accession number GSE108055.

### Cell lines

Two pulmonary carcinoids cell lines were used in this study at low passages (3 to 8). UMC-11 and NCI-H727 were obtained from American Type Culture Collection (ATCC, Manassas, VA). Cells were cultured according to ATCC recommendations, in RPMI-1640 medium (Lonza, Basel, Switzerland), supplemented with fetal bovine serum at 10% (FBS; Sigma-Aldrich, Madrid, Spain), L-glutamine at 1% (Sigma-Aldrich) and antibiotic/ antimycotic at 0.2% (Gentamicin/ Amphotericin B; Life Technologies). Both cell lines were checked monthly for mycoplasma contamination by PCR [26].

### RNA isolation, reverse transcription, qPCR and microfluidic qPCR array

Total RNA was isolated from the formalin-fixed paraffin embedded (FFPE) samples using Maxwell MDx 16 Instrument (Promega, Madrid, Spain) with the Maxwell 16 LEVRNA FFPE Kit (Promega, Madison, WI, USA), following manufacturer’s instructions. Total RNA from cell lines was extracted using the TRIzol/chloroform method (ThermoFisher-Scientific, Madrid, Spain). In both cases, isolated RNA was DNAse treated and quantified using Nanodrop One Microvolume UV–Vis Spectrophotometer (ThermoFisher-Scientific). RNA was retrotranscribed to copy DNA (cDNA) using random hexamer primers with RevertAid RT Reverse Transcription Kit (ThermoFisher-Scientific, #K1691).

Gene expression levels of target genes in FFPE samples were evaluated using a quantitative Real-Time PCR (qPCR) array based on microfluidic technology, using the Biomark System and the Fluidigm Real-Time PCR Analysis Software (Fluidigm, San Francisco, CA). To this end, specific primers for 43 components of the splicing machinery were specifically designed with Primer3 and Primer Blast software. These genes were selected based on their role on cancer, according to bibliographic information and our extensive previous experience [20, 22, 25, 27–30]. We adjusted RNA levels with three control genes (*ACTB*, *GAPDH* and *HPRT1*) using the geNorm software [31].

For cell lines studies, qPCR was used to measure gene expression, using 50 ng of cDNA and the Brilliant III SYBR Green Master Mix (Stratagene, La Jolla, CA) in the Stratagene Mx3000p system, as previously described by our group [20, 22]. Gene expression was normalized using *ACTB* gene, which levels were reproducibly stable across samples and did not differ between compared groups.

### Immunohistochemistry

To validate the presence of the proteins for the transcripts of interest, we examined a representative subset of 10 human samples, 8 typical carcinoids and 2 atypical carcinoids. Samples were fixed with formaldehyde 10% solution and embedded in paraffin, 5-µm sections obtained from FFPE samples were mounted in slides and were incubated with the primary antibody at 1:100 dilution, overnight (NOVA1, HPA004155, Sigma-Aldrich, Madrid, Spain; PRPF8, ab79237, Abcam, Cambridge, UK; SRSF1, PA5-30,220, ThermoFisher-Scientific; SRSF9, CSB-PA00214A0Rb, Cusabio Technology LLC, Houston, TX, USA; SRSF10, ab254935, Abcam). This was followed by incubation with anti-rabbit horseradish peroxidase at 1:250 dilution (#7074; Cell Signaling, Danvers, MA, USA) and slides were contrasted with hematoxylin/eosin stain. Percentage of positive cells and the staining intensity were evaluated by expert pathologists.

### Silencing of splicing factors in vitro

UMC-11 and NCI-H727 cell lines were transiently transfected with siRNAs to specifically knockdown the expression of *NOVA1* (#SR303213, OriGene, Rockville, MD, USA), *PRPF8* (#s20796, ThermoFisher-Scientific) and *SRSF10* (#s21157, ThermoFisher-Scientific). As a control, cells were transfected with Silencer Select Negative Control siRNA (ThermoFisher-Scientific). To this end, 350,000 cells/well were seeded in 6-well plates and transfected with 50 nM (*NOVA1*) or 75 nM (*PRPF8*, *SRSF10*) siRNAs using lipofectamine RNAiMAX reagent (ThermoFisher-Scientific) at 37 °C, following manufacturer’s instructions.

### Proliferation assay

To measure the effect of gene silencing on cell proliferation, resazurin (Canvax Biotech S.L., Córdoba, Spain) assay was used. Briefly, 5,000 transfected cells/well were seeded in a 96-well plate and serum-starved for 12 h. Resazurin (10%) was then added and fluorescence measured after 3 h of incubation with FlexStation III (Molecular Devices, San José, CA, USA) at 0, 24, 48, 72 and 96 h of culture.

### Colony formation assay

Colony formation capacity was evaluated after gene silencing. For this purpose, 5,000 transfected cells/well were seeded in 6-well plates. After 10 days of culture in complete medium, changing medium every 3 days, cells were fixed and stained with crystal violet (0.5%) and glutaraldehyde (6%) solution and analyzed using Fiji [32].

### Western blot

Western blot analyses were performed to check protein levels in cell lines. Briefly, cells were transfected with either siRNA or scramble, the culture medium was aspirated, and 300 μl of a pre-warmed SDS-DTT solution at 65 °C was added to lyse the cells. Subsequently, the samples were sonicated for 10 s and boiled for 5 min at 95 °C. Protein samples were then separated on 12.5% polyacrylamide gels through SDS-PAGE, followed by transfer onto a nitrocellulose membrane (Millipore, #1704270). The membrane was then blocked using a solution of 5% non-fat dry milk in Tris-buffered saline containing 0.05% Tween-20 (Sigma-Aldrich, #93773). Next, the membranes were incubated with specific primary antibodies, including NOVA1 (Abcam, #ab183024), PRPF8 (Abcam, #ab79237), and SRSF10 (Abcam, #ab254935). Then, they were incubated with secondary anti-rabbit antibody (Cell Signaling, #7074S). Antibodies intensity was visualized using the Clarity Western-ECL Blotting Substrate from Bio-Rad Laboratories (Madrid, Spain), and the resulting blots were scanned with an ImageQuant Las 4000 system (GE Healthcare Europe GmbH). Images were analyzed using ImageJ-1.51 s software.

### Bioinformatic and statistical analyses

To further study the molecular profile of pulmonary carcinoids, we explored a cohort of 20 atypical carcinoids with available RNA-seq data (dataset EGAD00010001719 from the European Genome-Phenome Archive), from which the two samples labelled as supracarcinoids were removed from analysis because of their distinct molecular profile [8]. To this aim, paired FASTQ files were pseudo-aligned with Salmon [33], using the v34 version of the human transcriptome annotation (GENCODE). The quantification files were imported into R with tximeta package [34] and counts were normalized with DESeq2 using the variance-stabilization transform [35]. The Gene Set Enrichment Analysis (GSEA) software [36] was used for enrichment analysis using normalized gene expression. For alternative splicing studies, transcript per million (TPM) from Salmon quantification files were used to calculate the Percent Spliced In (PSI) from alternative splicing events of the whole transcriptome using SUPPA2 [37]. Differences in PSI between groups were calculated using SUPPA2 empirical testing and those events with *p* < 0.05 were considered significantly different.

For gene expression quantification, data are represented as mean ± standard error of the mean (SEM), or relative levels in comparison with control. Kolmogorov–Smirnov test was performed to check for normality of data and, consequently, Student *t* (parametric) or Wilcoxon (non-parametric) tests were applied to test for differences. To identify the ability of the different variables measured to discriminate between tumor and non-tumor tissue, Partial Least Squares Discriminant Analysis (PLSDA) was used. VIP (Variable Importance in Prediction) Score analyses were performed to identify the variables with the highest contribution to the PLSDA generated model. Both PLSDA and VIP Scores were performed using MetaboAnalyst 5.0 [38]. Three different replicates of the in vitro experiments were carried out. Statistical significance was set at *p* < 0.05. Statistical analyses were performed using Prism v8.0 (GraphPad, La Jolla, CA, USA), R v4.0.4 and RStudio software v1.3.1093.

## Results

### Expression profile of the splicing machinery is altered in pulmonary carcinoids, enables to discriminate tumor vs. non-tumor tissue, and unveils new molecular links with clinical features.

**Fig. 1:**
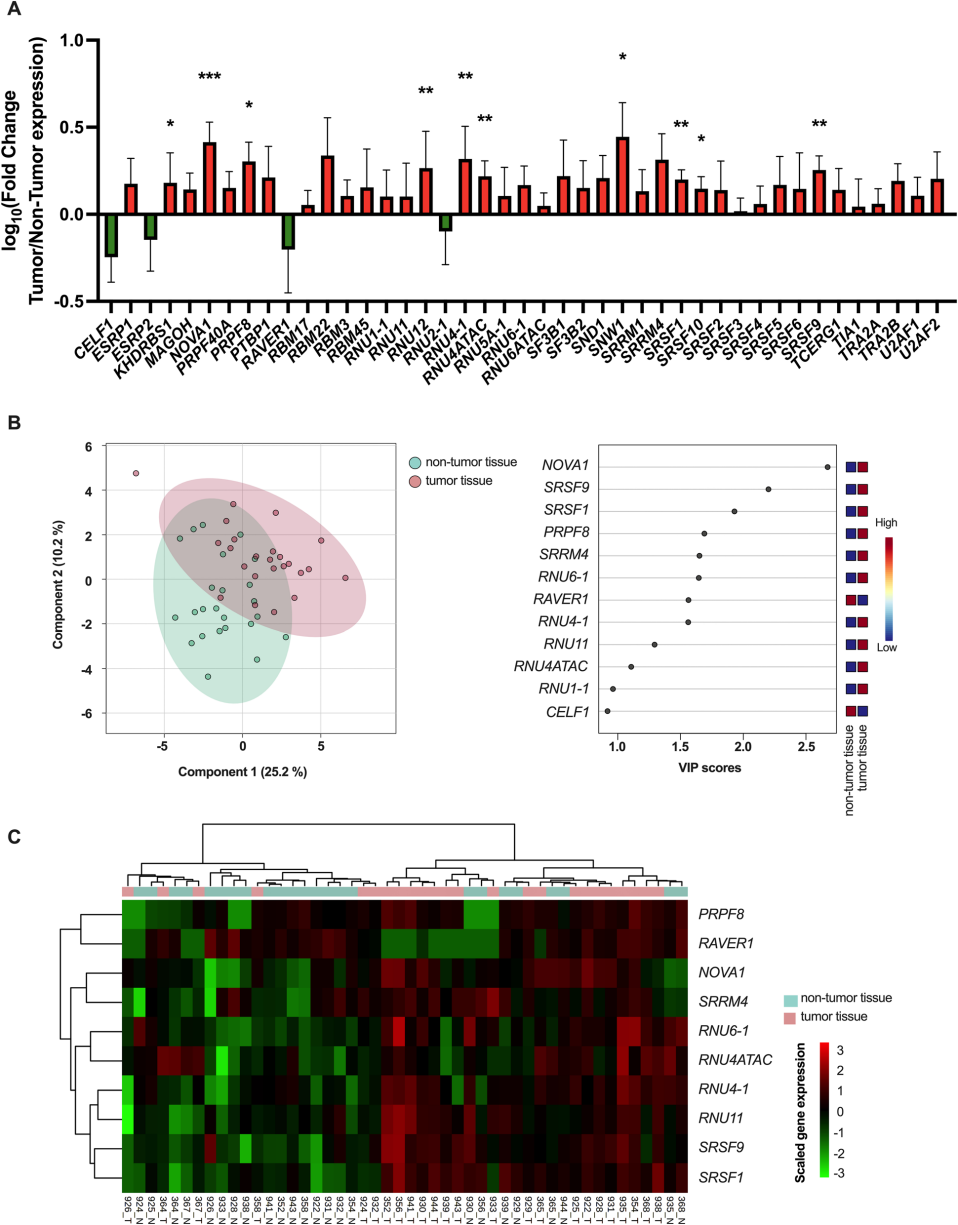
The splicing machinery is profoundly dysregulated in lung carcinoids. **A**. Individual fold-change of the RNA expression levels of all the splicing machinery components analyzed in lung carcinoids FFPE samples [n = 23 (typical carcinoids, atypical carcinoids, and undetermined carcinoids)] compared with non-tumoral adjacent tissue [n = 24]. Data represents mean ± SEM. Asterisks indicate significant differences (* p < 0.05; **p < 0.01; ***p < 0.001). **B**. Partial least squares discriminant analysis (PLSDA) of the RNA expression levels of the splicing machinery components in the Discovery cohort. VIP scores obtained from PLSDA of the complete splicing machinery studied. **C.** Hierarchical heatmap generated with the expression levels of the top 10 genes of the splicing machinery that contribute most to the discrimination between tumor tissue (red) and adjacent non-tumor tissue (green)

The expression of 10 of the 43 splicing machinery components evaluated (23.3%) was altered in pulmonary carcinoid tissue when compared to their respective non-tumor adjacent tissue (Wilcoxon test, *p* < 0.05; Fig. 1A). Specifically, the splicing machinery components *KHDRBS1*, *NOVA1*, *PRPF8*, *SNW1*, *SRSF1*, *SRSF10* and *SRSF9* were overexpressed in tumor tissue. Moreover, in the core of the spliceosome machinery, the snRNAs *RNU4-1,* of the major spliceosome, and *RNU12* and *RNU4ATAC,* of the minor spliceosome, were also overexpressed in tumor tissue. No overt differences were observed between the two carcinoid subtypes (see Histology in Additional file 2: Figure S2). To examine these results in more detail, we analyzed an external validation cohort (GSE108055, Additional file 1: Figure S1) [39]. The dataset explored in this case derives from a mRNA expression microarray, which contains nearly 80% of the genes evaluated in our microfluidic array (34 out of 43 genes), mostly because snRNA (which lack a poly-A tail) were not targeted by this technique. Interestingly, 16 of the 34 components examined (47.1%) were altered and, in line with our Discovery cohort, *KHDRBS1*, *NOVA1*, *PRPF8*, *SNW1*, *SRSF1*, and *SRSF9* were also overexpressed in tumor tissue in this external cohort (Additional file 1: Figure S1A). At this point, to analyze these results with a more objective perspective, we should introduce the caveat that the wide diversity of cell types in the tumor surrounding tissue, together with the low proportion of neuroendocrine cells in bronchial tissue [40] is admittedly a general limitation in the study of these tumors, as it hinders a balanced comparison between the tumor tissue and the adjacent non-tumor component of the tissue. Hence, we routinely consider the neighboring non-tumor tissue more as a reference tissue for comparisons than a bona fide control tissue. Notwithstanding this, the caveat does not preclude comparing both tissues, and, therefore, we applied a customized biocomputational and statistical approach developed for this purpose [41]. Specifically, partial least squares discriminant analysis (PLSDA) of the expression data revealed that splicing-related genes were good discriminators of tumor vs non-tumor tissue. Moreover, the Variable Importance in Projection (VIP) Scores allowed to quantify the importance of each splicing-related gene to the discriminant model (Fig. 1B). The application of the same type of analysis to the external validation cohort resulted in a highly similar outcome, in that the expression levels of the splicing-related genes clearly discriminated tumor from non-tumoral tissue and both VIP Scores models displayed a substantial overlap with 5 shared genes (Additional file 1: Figure S1B). In line with these observations, non-supervised hierarchical clustering using the top 10 discriminant genes according to VIP Scores unveiled two major clusters that were respectively enriched (Fisher’s exact test *p* = 0.004) in non-tumor and tumor samples (Fig. 1C, Additional file 1: Figure S1C).

**Fig. 2:**
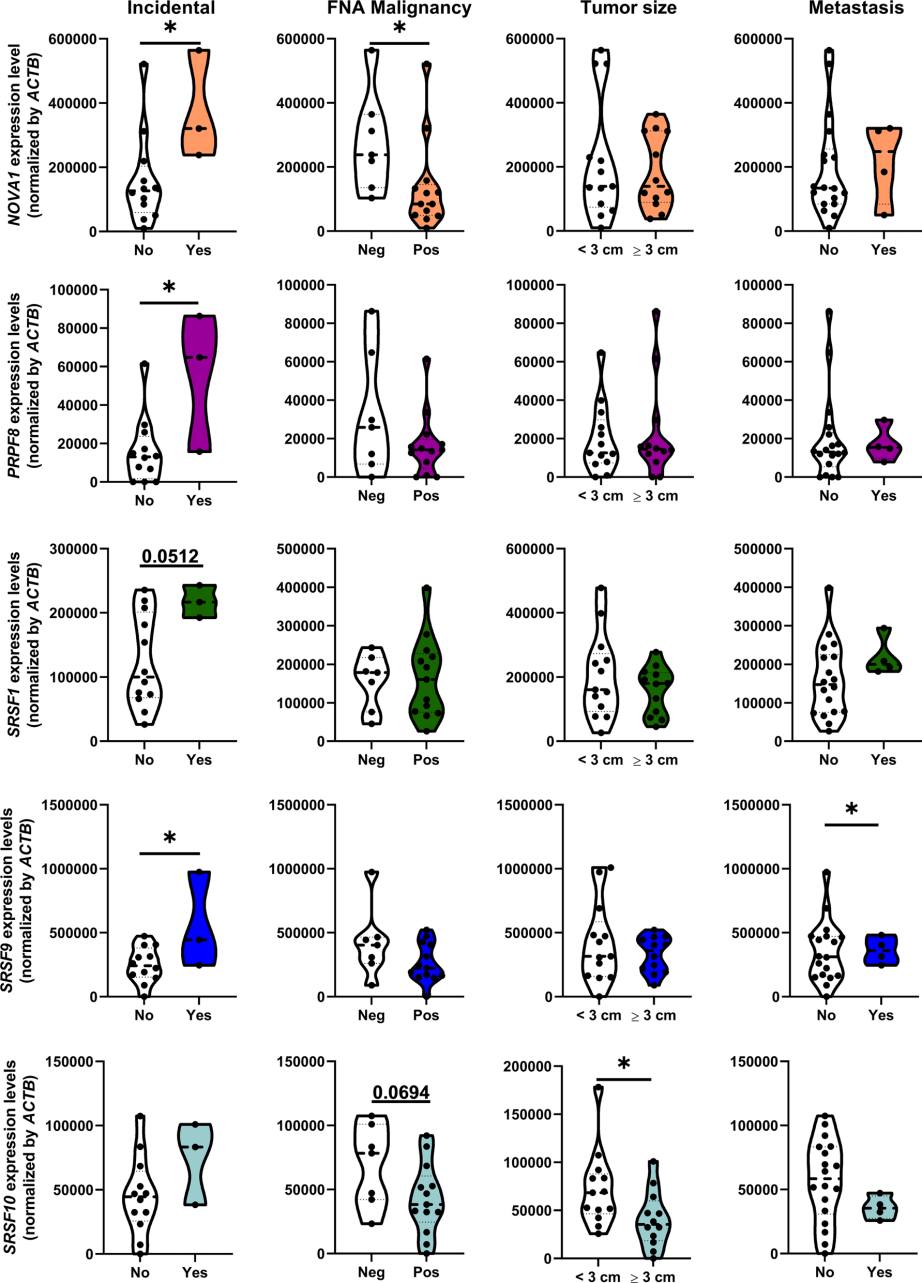
Association of splicing machinery dysregulation with key clinical parameters in lung carcinoids from the Discovery cohort. Correlation of selected splicing factors mRNA levels with incidental diagnosis, FNA malignancy, diameter, and metastasis in the Discovery cohort. Data represents mean ± SEM. Asterisks indicate significant differences (*p < 0.05)

Based on PLSDA and clustering analysis, we selected the top four dysregulated components of the splicing machinery displaying the best discriminating capacity to further explore their role in pulmonary carcinoids, namely: *NOVA1*, *PRPF8*, *SRSF1* and *SRSF9*. Of note, these genes were also among the best discriminators of the PLSDA analysis in the validation cohort. Simultaneously, a global screening of the potential associations between the expression levels of each of the splicing factors measured with the most relevant clinical parameters of patients provided an informative snapshot (Additional file 2: Figure S2), which allowed us to select another interesting component of the splicing machinery, *SRSF10,* that was also overexpressed in tumor tissue. As illustrated in Fig. 2, these five genes showed similar association profile between their increased expression and incidental diagnosis, reaching statistical significance for *NOVA1*, *PRPF8* and *SRSF9*. In addition, *NOVA1* expression levels were lower when positive malignancy was confirmed after fine needle aspiration, while *SRSF9* expression was higher in metastatic disease. Moreover, *SRSF10* expression was negatively associated to tumor diameter.

### Protein levels of selected splicing factors unveil heterogeneous distribution in tumor tissue

**Fig. 3:**
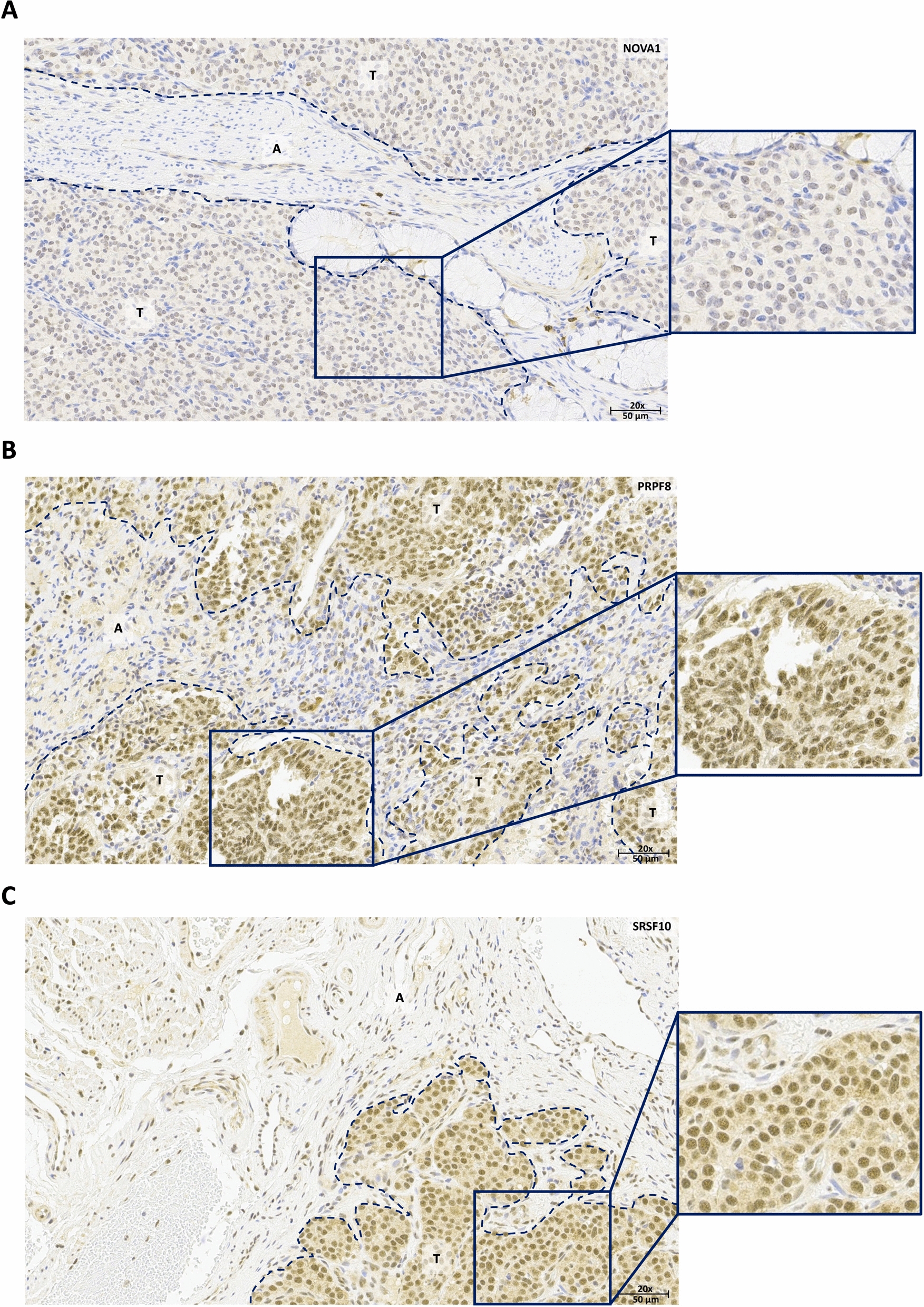
Representative IHC 20X-images from three carcinoids stained with NOVA1** (A)**, PRPF8 (**B**) and SRSF10 (**C**). Staining is more intense in tumor tissue with respect to adjacent non-tumor tissue in the cytoplasm for all three factors and in the nucleus for SRSF10. Scale bar indicates 50 µm

The presence of the selected splicing factors in carcinoids was further examined by IHC analysis, which confirmed that the protein of three splicing factors, NOVA1, PRPF8 and SRSF10 was detectable in tissue samples. In particular, NOVA1 exhibited a moderate focal cytoplasmic staining and intense but heterogeneous nuclear staining in tumor tissue (Fig. 3A), while, in the adjacent non-tumor tissue, composed of connective tissue and seromucous glands, an almost complete absence of staining was observed. In the case of SRSF10, the tumor tissue showed a mild staining at the cytoplasmic level that contrasted with an intense and uniform staining at the nuclear level, whereas adjacent non-tumor tissue showed very weak staining in the cytoplasm and weak and diffuse staining in the nuclear compartment (Fig. 3C). Likewise, IHC for PRPF8 revealed a moderate staining in the cytoplasmic compartment accompanied by intense staining at the nuclear level in the tumor component of the sample, similar to that described for SRSF10; in contrast, the adjacent non-tumor tissue showed weakly stained cytoplasm and nuclei lacking staining (Fig. 3B). Thus, in line with the RNA expression data, the IHC analysis revealed an overexpression of the three splicing factors NOVA1, PRPF8 and SRSF10. Conversely, application of a similar approach using various methods and antibodies did not reveal consistent differences in the signal abundance and intensity for SRSF1 and SRSF9 in tumor vs. non-tumor tissue.

### NOVA1, PRPF8 and SRSF10 have distinct molecular profiles associated to their expression

**Fig. 4:**
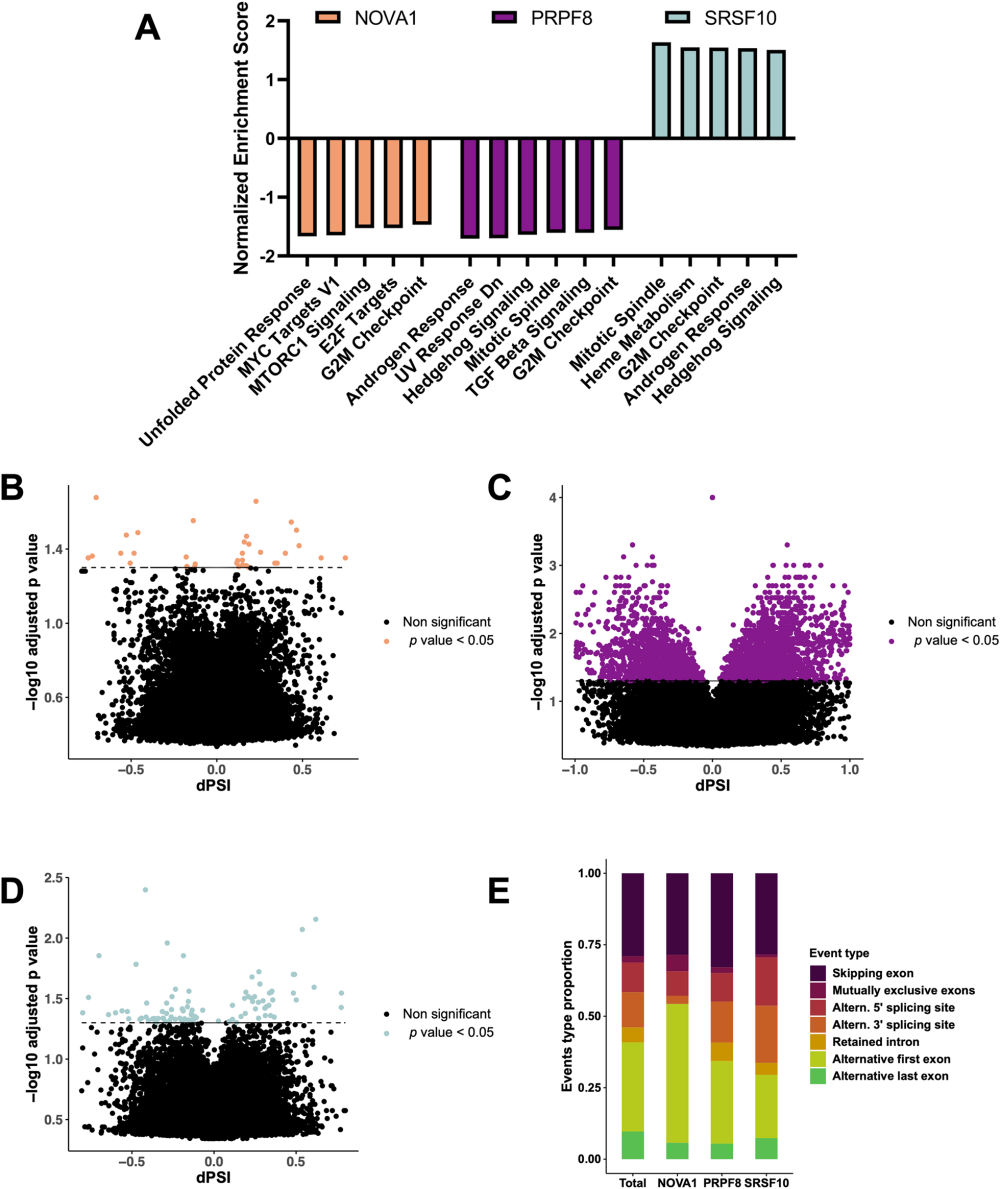
Molecular signatures associated to *NOVA1*, *PRPF8* and *SRSF10* expression in RNA-seq data from pulmonary carcinoids. **A**. Gene Set Enrichment Analysis (GSEA) using Hallmarks gene set to look for molecular pathways associated to *NOVA1*, *PRPF8* and *SRSF10* expression. Normalized Enrichment Score is represented for each of the pathways, being plotted only those pathways with *p* < 0.05. **B**, **C**, **D**. Volcano plots showing differential PSI of alternative splicing events against -log10 *p* value, when comparing high and low expression groups from *NOVA1* (**B**), *PRPF8* (**C**) and *SRSF10* (**D**). Only statistically significant events are colored (*p* < 0.05). **E**. Bar plot showing the proportion of each of the alternative splicing event’s patterns to which belong the total events, *NOVA1*, *PRPF8* and *SRSF10* associated events

To explore in more detail the potential role of *NOVA1*, *PRPF8* and *SRSF10* in pulmonary carcinoids, we analyzed a publicly available RNA-seq dataset (EGAD00010001719) from 18 atypical carcinoids. Gene set enrichment analysis (GSEA) performed according to Hallmarks gene sets revealed that the expression of each splicing factor distinctly correlated to a discrete number of hallmarks (Fig. 4A). Thus, whereas *NOVA1* was negatively correlated with genes belonging to unfolded protein response, MYC targets, MTORC1 signaling, E2F targets, and G2M checkpoint, the expression of *PRPF8* was negatively associated to androgen response, genes downregulated by UV response, Hedgehog signaling, mitotic spindle, TGF beta signaling and G2M checkpoint. In marked contrast, *SRSF10* expression was positively correlated to genes that belong to mitotic spindle, heme metabolism, G2M checkpoint, androgen response and Hedgehog signaling. Interestingly, some of the altered pathways, particularly G2M checkpoint, were shared across the three splicing factors.

Inasmuch as the primary known role for NOVA1, PRPF8 and SRSF10 is their function as splicing factors, we aimed at examining their putative relationship with the alternative splicing profile in carcinoid cells. To this end, we calculated the Percent Spliced In (PSI) of alternative splicing events in every tumor sample of the RNA-seq. Samples were classified according to the expression of each splicing factor into high and low expressing samples, and differences in alternative splicing were calculated between both groups. This approach allowed us to assess the potential association between the expression levels of each splicing factor and the pattern of alternative splicing inside the tumor, which could bear functional implications. Interestingly, results unveiled very distinct association patterns for each of the studied factors. Specifically, whereas *NOVA1* displayed a reduced set of 35 significantly altered alternative splicing events associated to its low/high expression level (Fig. 4B), the expression of *PRPF8* was associated to 2905 significant events (Fig. 4C), and that of *SRSF10* to 95 events (Fig. 4D). Differences among splicing factors are not related only to the number but also to the distinct patterns of alternative splicing associated to each of them. Thus, as illustrated in Fig. 4E, whereas *NOVA1* was associated to less intron retaining, and more alternative first exon events, *PRPF8* displayed an increase of skipping exon events and a clear reduction of first and last exon events, and *SRSF10* associated events were enriched in 5’ and 3’ alternative splice sites to the detriment of alternative first exon events.

### Targeting splicing factors in vitro elicits antitumoral effects in lung carcinoid cell models

**Fig. 5:**
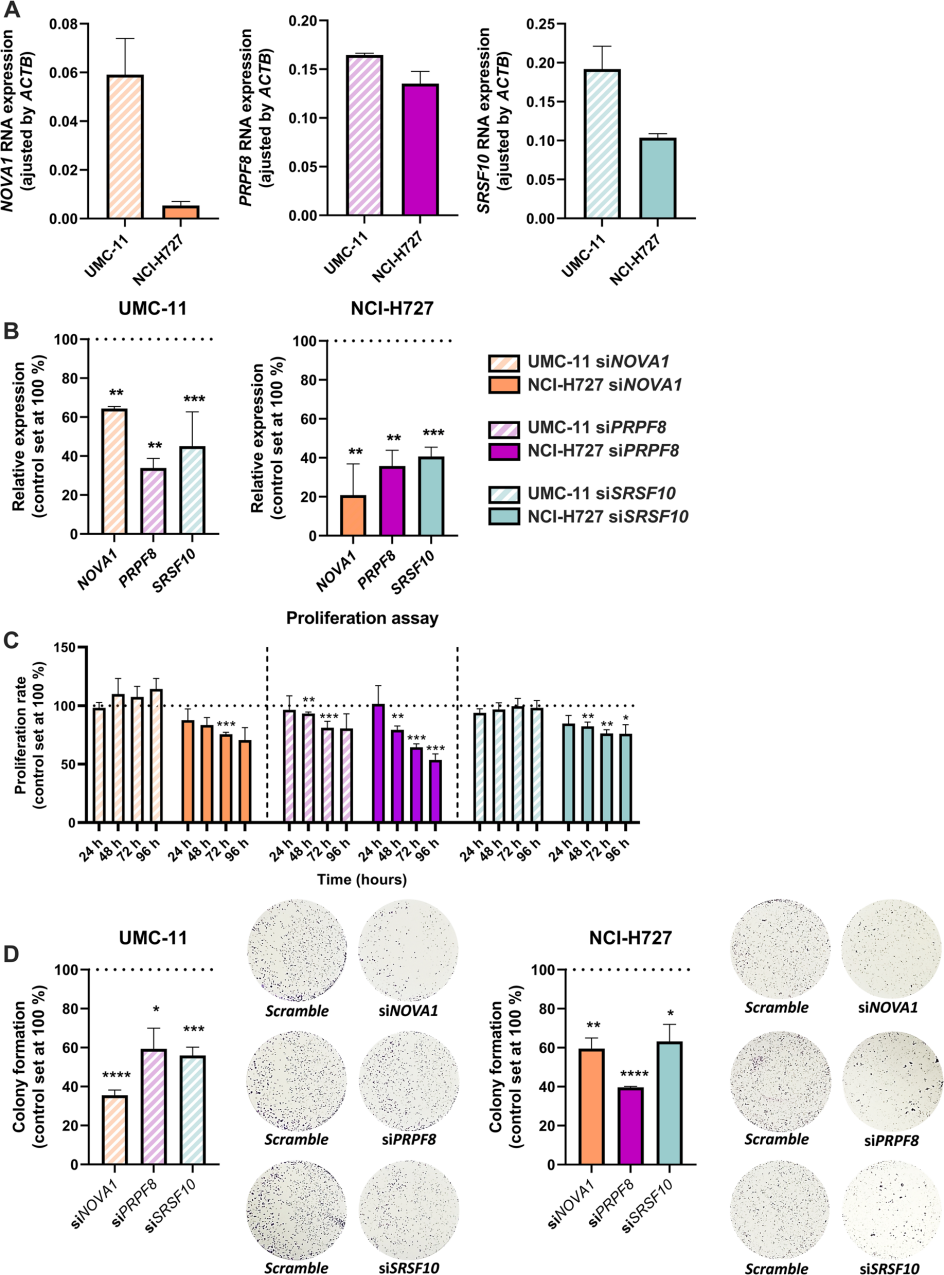
Effect of *NOVA1*, *PRPF8* and *SRSF10* modulation on lung carcinoid cell lines. **A**. *NOVA1*, *PRPF8* and *SRSF10* basal expression levels in UMC-11 and NCI-H727 cell lines adjusted by *ACTB* expression levels. **B**. Validation of *NOVA1*, *PRPF8* and *SRSF10* silencing in lung carcinoid cell lines by qPCR. Data are expressed as a mean ± SEM as percentage of control (scramble; set at 100%) (n = 3). **C**. Proliferation rate of *NOVA1*, *PRPF8* and *SRSF10*-silenced cells compared to control scramble-transfected lung carcinoid cells (n = 3). **D**. Colony formation capacity of *NOVA1*, *PRPF8* and *SRSF10*-silenced cells compared to control scramble-transfected lung carcinoid cells (scramble; set as 100%). Representative images of colony formation (n = 3)

Having shown the alternative splicing-related features associated to each splicing factor, we next aimed to interrogate the possible functional role played by these factors in pulmonary carcinoids. To this end, since their expression was augmented in tumor tissue, we performed silencing experiments of *NOVA1*, *PRPF8* and *SRSF10* in UMC-11 and NCI-H727 cells, two distinct broadly used pulmonary carcinoid cell models (Fig. 5). We first found that, despite their varied levels of expression under basal culture conditions, the silencing of the three factors was comparably effective in each cell line, being overall more pronounced in NCI-H727 with respect to UMC-11 cells (Fig. 5A, B). Basal and protein levels after silencing were also validated using Western blot (Additional file 3: Figure S3). Silencing *NOVA1* and *SRSF10* decreased NCI-H727 cell proliferation at 72 h and at 48, 72 and 96 h, respectively, when compared to scrambled-transfected cells. However, no effects on cell proliferation were detected in UMC-11 cell line (Fig. 5C). Meanwhile, silencing *PRPF8* showed a marked decrease on cell proliferation in both cell lines after 48 h of expression inhibition. Moreover, *NOVA1*, *PRPF8* and *SRSF10* silencing also decreased colony formation ability of both UMC-11 and NCI-H727 cell lines, being *NOVA1* silencing the one that exerted the highest effect on UMC-11 cells and *PRPF8* silencing in NCI-H727 cells (Fig. 5D).

## Discussion

Pulmonary carcinoids are well differentiated neuroendocrine neoplasms with rising incidence [42]. While their molecular landscape is progressively being deciphered in recent years [8–10], its precise role in tumor biology is still poorly understood and its clinical translation awaits to be exploited [11–13]. Alternative splicing dysregulation is a hallmark common to many cancers [17], including neuroendocrine neoplasms [21]. Indeed, altered splicing can contribute to tumor initiation, progression and drug response by altering the pattern of splicing of many genes, thereby causing the loss of essential variants for cell homeostasis and appearance of aberrant oncogenic splice variants [17, 21, 43]. A leading cause for such alterations resides in mutations and altered expression in splicing machinery components, which can modify both global patterns of splicing and the set of variants of specific genes [44]. In this study, we explored the status of the splicing machinery in pulmonary carcinoids and identified a set of key components altered in tumor vs. non-tumor adjacent tissue, which are linked to clinico-pathological features and exert functionally relevant roles in cell models, suggesting their potential as tools to develop new biomarkers and actionable targets for this rare disease.

The differences observed in the expression profile of the splicing machinery in pulmonary carcinoid tissue and its surrounding non-neoplastic tissue was expectable, in line to that observed by our group and others in various types of cancer, including pancreatic neuroendocrine tumors [21, 23, 25, 45–48]. Unlike in other cancers (e.g. prostate, liver), a proper comparison between normal and tumor neuroendocrine cells in carcinoids is precluded by the fact that normal neuroendocrine cells only comprise 0.4% of lung airway epithelial cells [40]. Notwithstanding this limitation, the differences found do illustrate that tumor tissue displays a distinct splicing machinery landscape. Actually, in keeping with that found in most tumors (except for pituitary adenomas) [21, 23, 25, 45–47], the altered components showed higher expression in tumor tissue than in non-tumor tissue, inviting to further explore these molecules as potential diagnostic and prognostic biomarkers. In this regard, selection of the best candidates to be studied in detail can benefit from a two-pronged approach combining objective biocomputational scoring [41] and assessing their association with clinically relevant parameters. Application of this bioinformatic strategy selected four factors for further analysis: *NOVA1*, *PRPF8*, *SRSF1* and *SRSF9*, while clinical association with tumor size suggested an additional candidate, *SRSF10*. The inclusion of *SRSF10* in the study was also motivated by its well described pro-malignant role in other tumors [49, 50]. Indeed, splicing factor overexpression in tumors commonly results in altered splicing patterns, which can be linked to pathological outcomes. Therefore, finding correlations between splicing factor expression and clinical parameters could guide to relevant discoveries [21, 22, 25, 30, 45, 47]. Accordingly, not only *SRSF10* expression but that of the other four candidates displayed associations with important diagnostic parameters, such as incidental diagnosis, detection of malignancy using fine needle aspiration or tumor size, highlighting their potential as diagnostic biomarkers. Although, the implications of these discoveries may not always be evident. Thus, on the one hand, higher expression of several splicing factors in incidental diagnosis may suggest a possible relation of their dysregulation with a more difficult detection of the tumors by currently employed screening methods, thereby suggesting that their elevated levels could provide an opportunity to explore new biomarkers for early detection in the future. On the other hand, the finding of lower *NOVA1* expression levels being linked to malignancy detection in FNA is somewhat counterintuitive, since, given its higher levels in tumor tissue compared with surrounding non-tumor tissue, one could expect higher levels when malignancy is detected by FNA. Besides technical considerations inherent to FNA (e.g. restricted anatomical tumor location, variability, etc.) we could not find a sound explanation to understand this intriguing observation, which, obviously, will require further work.

Further analysis of the potential of the selected splicing factors as valuable molecular candidates involved the assessment of their actual presence as proteins in the tumor, their putative association to the predicted role as modulators of splicing, and their requisite nature as actionable targets, i.e., their ability to play a relevant functional role in suitable models. Testing the first of these criteria revealed that not all the overexpressed splicing factors found by RNA measurements could be confirmed by pathological inspection of immunohistochemical staining in tumor samples, either due to technical limitations or by a true quantitative discrepancy between the amount of mRNA and protein present in the tumors. This approach reduced the number of candidates considered more suitable to serve as biomarker and targetable tools, i.e., *NOVA1*, *PRPF8* and *SRSF10*.

Analysis of the expression of these three factors already revealed their association to distinct key molecular pathways, such as cell cycle-related or cell signaling-related processes. Particularly, cell cycle genes, which have been closely linked to the three factors studied, are quite relevant in pulmonary carcinoids, as they define the mitotic rate and therefore the grade and prognosis of the tumors [1]. Previous studies have used transcriptomic analyses to identify mitotic rate of pulmonary carcinoids and refine their classification [51]. Of similar importance, cell signaling-related processes are also known to be essential to understand pulmonary carcinoids biology. Among the most relevant and studied pathways are the mTOR signaling cascade, which is frequently mutated, and stands as a widely recognized treatment target in these tumors [52], and the TGF-β signaling pathway, whose components are also frequently altered [53]. In contrast, Hedgehog and androgen signaling are not so well studied in pulmonary carcinoids but are known to be involved in lung differentiation [54, 55].

Further bioinformatic approaches enabled to explore the putative relationship of these splicing factors with molecular parameters informing on the result of the splicing process. Interestingly, examination of various datasets revealed that the expression levels of *NOVA1*, *PRPF8* and *SRSF10* were differentially, but consistently linked to genuine divergencies in the pattern of splicing events in cohorts of well differentiated carcinoids, lending credence to our prediction that their overexpression could be linked to altered splicing in these tumors. In particular, *PRPF8* expression was linked to a remarkable number of significantly altered alternative splicing events, which is likely related to its important role in the spliceosome structure, where it takes part as member of the main core. In contrast, *NOVA1* and *SRSF10* expression were linked to a more limited number of splicing events, supporting the contention that these 3 altered factors may be involved in relevant but distinct functional roles in carcinoids.

To test the above notion, we developed functional assays using carcinoid model cell lines, which clearly demonstrated that the alterations identified in the selected splicing factors can lead to changes in functional features of the tumor cells, such as cell proliferation or colony formation. Interestingly, these analyses also revealed informative differences among splicing factors and across cell lines. Thus, whereas silencing of PRPF8, NOVA1 and SRSF10 comparably reduced both UMC-11 and NCI-H727 cells colony formation, this silencing reduced cell proliferation more consistently in NCI-H727 cells, whereas, in contrast, in the UMC-11 line, only *PRPF8* silencing seemed to reduce cell proliferation. These results unveil subtle, previously unrecognized differences between the behavior of the two cell lines in relation to splicing factor function, providing experimental support to our proposal that the three factors could play distinct roles in carcinoids. Of note, the main difference between the two cell lines lies in the resistance to treatment by the UMC-11 line, a characteristic that does not appear in the NCI-H727 line [56].

Future studies should aim to unravel the molecular determinants underlying the different roles of these factors and their possible relationship with treatment resistance, as well as to overcome the limitations of the present study. Besides the abovementioned limitation posed by the use of surrounding non-tumoral tissue as a reference, our study has an intrinsic limitation in the modest number of samples analyzed, which we somehow circumvented by studying validation cohorts from external sources. Nevertheless, it is clear that in order to provide further support to our findings and to achieve a deeper understanding of their implications, it would be ideal to analyze in more detail a larger tumor cohort, with a higher number of more representative samples of lung carcinoids, and studies are already ongoing aimed to that goal. This would also allow to face new challenges that are prompted by the results discovered in the present work, namely, the elucidation of the molecular underpinnings causing the dysregulation of the splicing machinery, and the molecular, functional and pathological consequences of the changes observed. Thus, one of the most obvious future challenges is the analysis of the specific molecules, the splice variants, that are altered as a consequence of the dysregulated splicing factors, and the elucidation of their putative contribution to the functional consequences observed. As well, this will lead to investigate the pathological consequences involved by the changes in those molecular players, as well as their potential translational significance.

The present discovery that these factors could contribute to the development and/or progression of lung carcinoids is in line with available data on other tumors, which further substantiates the idea that their independent silencing can hinder carcinoid growth. In fact, an increasing number of studies shows that targeting some of these factors can have antitumor properties, as in the case of *NOVA1* in non-small cell lung cancer [57], pancreatic neuroendocrine tumors [20], osteosarcoma [58] and astrocytoma [59]; or *SRSF10* in colon cancer [50], hepatocarcinoma [60] or head and neck cancer [49]. Moreover, in line with the robust inhibitory effects observed after silencing *PRPF8*, this core component of the major spliceosome has already been associated to malignancy in prostate cancer [61], hepatocarcinoma [62, 63] and breast cancer [64].

## Conclusions

In summary, our work primarily unveils a clear alteration of the splicing machinery in lung carcinoids that is linked to three specific factors, NOVA1, PRPF8 and SRSF10, which are differentially associated to pathological features, distinct profiles of splicing events, and key functional actions. These findings underscore the potential of the splicing machinery, and the splicing process at large, as a novel source to better understand tumor biology and to identify candidate biomarkers and actionable targets. Thus, the role of these three factors as putative oncogenes for tumor development and aggressive behavior in lung carcinoids warrants further study.

### Supplementary Information

**Additional file 1: Figure S1A.** RNA expression levels of all the splicing machinery components analyzed in lung carcinoids frozen samples [n = 42 (typical carcinoids and atypical carcinoids)] compared with non-tumoral adjacent tissue samples (n = 9) in the external cohort (GSE108055). **B.** PLSDA of the RNA expression levels of the splicing machinery components in the validation cohort (top). VIP scores obtained from PLSDA of the complete splicing machinery studied (bottom). **C.** Hierarchical heatmap generated with the expression levels of the top 12 genes of the splicing machinery that contribute most to the discrimination between tumor tissue (red) and adjacent non-tumor tissue (green) in the validation cohort

**Additional file 2: Figure S2.** Association of the expression levels of components of the splicing machinery with different relevant clinical parameters in the Discovery cohort. The size of the circles refers to the *p* value of the clinical association

**Additional file 3: Figure S3A.** Protein levels of NOVA1, PRPF8 and SRSF10 in model cell lines under basal conditions (n = 3) as assessed by Western Blot analysis. **B.** Protein levels of NOVA1, PRPF8 and SRSF10 in model cell lines after respective gene silencing using specific siRNA (n = 3). Data were normalized with Ponceau and represented as percentage compared to Scramble (set at 100%). Data represents mean ± SD. Asterisks indicate values that significantly differences between groups (*, p < 0.05; **, p < 0.01; ****, p < 0.0001)

## Data Availability

The discovery cohort dataset used and analyzed during the current study is available from the corresponding author on reasonable request. The validation cohorts analyzed in this study are available in Gene Expression Omnibus (GEO; under accession number GSE108055) and in European Genome-Phenome Archive (under accession number EGAD00010001719).

## Abbreviations

LungNENs: Lung Neuroendocrine Neoplasms
LCNEC: Large Cell Neuroendocrine Carcinoma
SCLC: Small Cell Lung Cancer
FFPE: Formalin-fixed Paraffin Embedded
PSI: Percent Spliced In
PLSDA: Partial Least Squares Discriminant Analysis
VIP: Variable Importance in Projection
