# Supplementary figures and images for: Altered splicing machinery in lung carcinoids unveils NOVA1, PRPF8 and SRSF10 as novel candidates to understand tumor biology and expand biomarker discovery
Source: J Transl Med. 2023 Dec 4;21:879. doi: 10.1186/s12967-023-04754-8 (PMC10696873; doi:10.1186/s12967-023-04754-8)

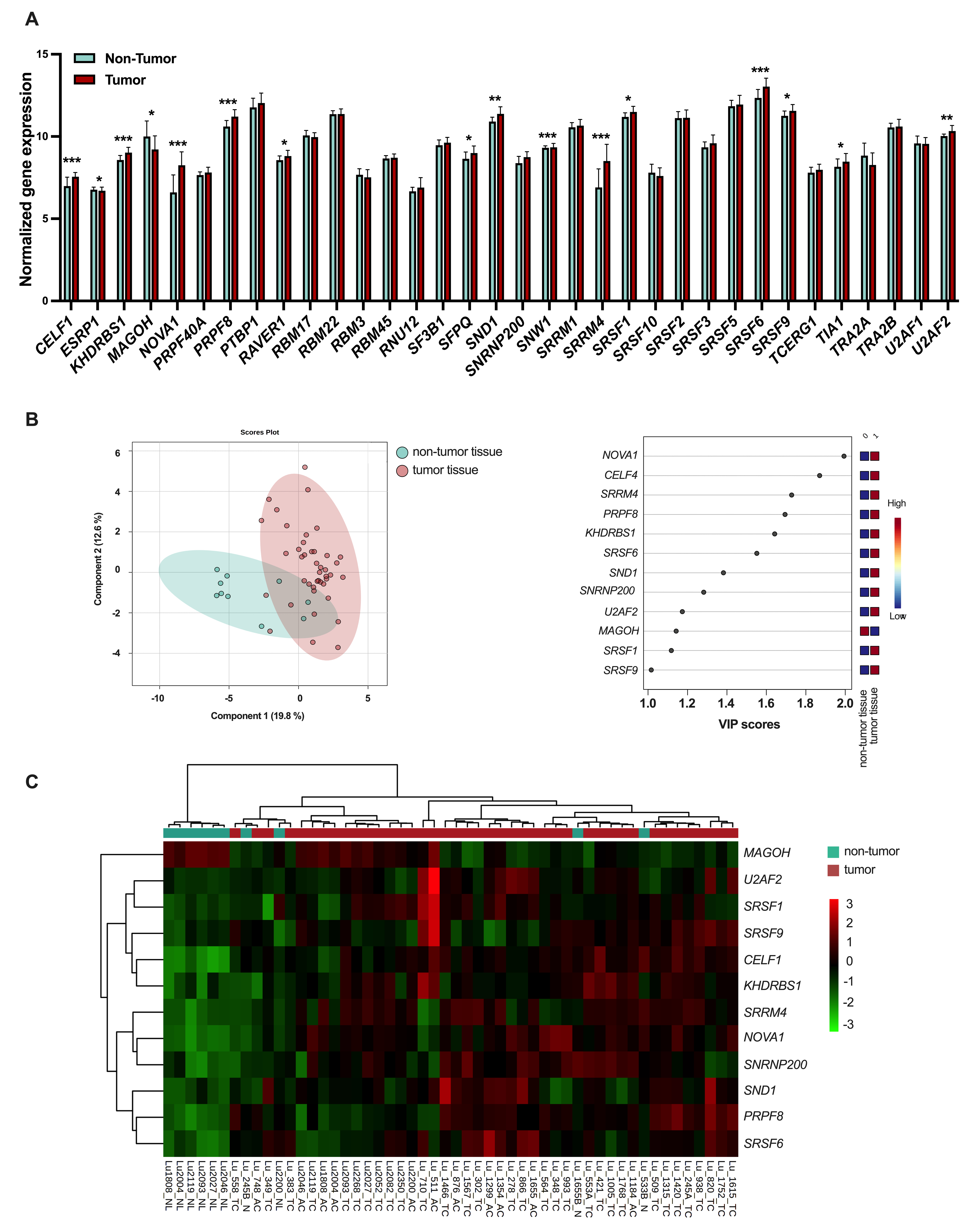

Supplement: Supplementary file 1 — Additional file 1: Figure S1A. RNA expression levels of all the splicing machinery components analyzed in lung carcinoids frozen samples [n = 42 (typical carcinoids and atypical carcinoids)] compared with non-tumoral adjacent tissue samples (n = 9) in the external cohort (GSE108055). B. PLSDA of the RNA expression levels of the splicing machinery components in the validation cohort (top). VIP scores obtained from PLSDA of the complete splicing machinery studied (bottom). C. Hierarchical heatmap generated with the expression levels of the top 12 genes of the splicing machinery that contribute most to the discrimination between tumor tissue (red) and adjacent non-tumor tissue (green) in the validation cohort [file 12967_2023_4754_MOESM1_ESM.jpg]

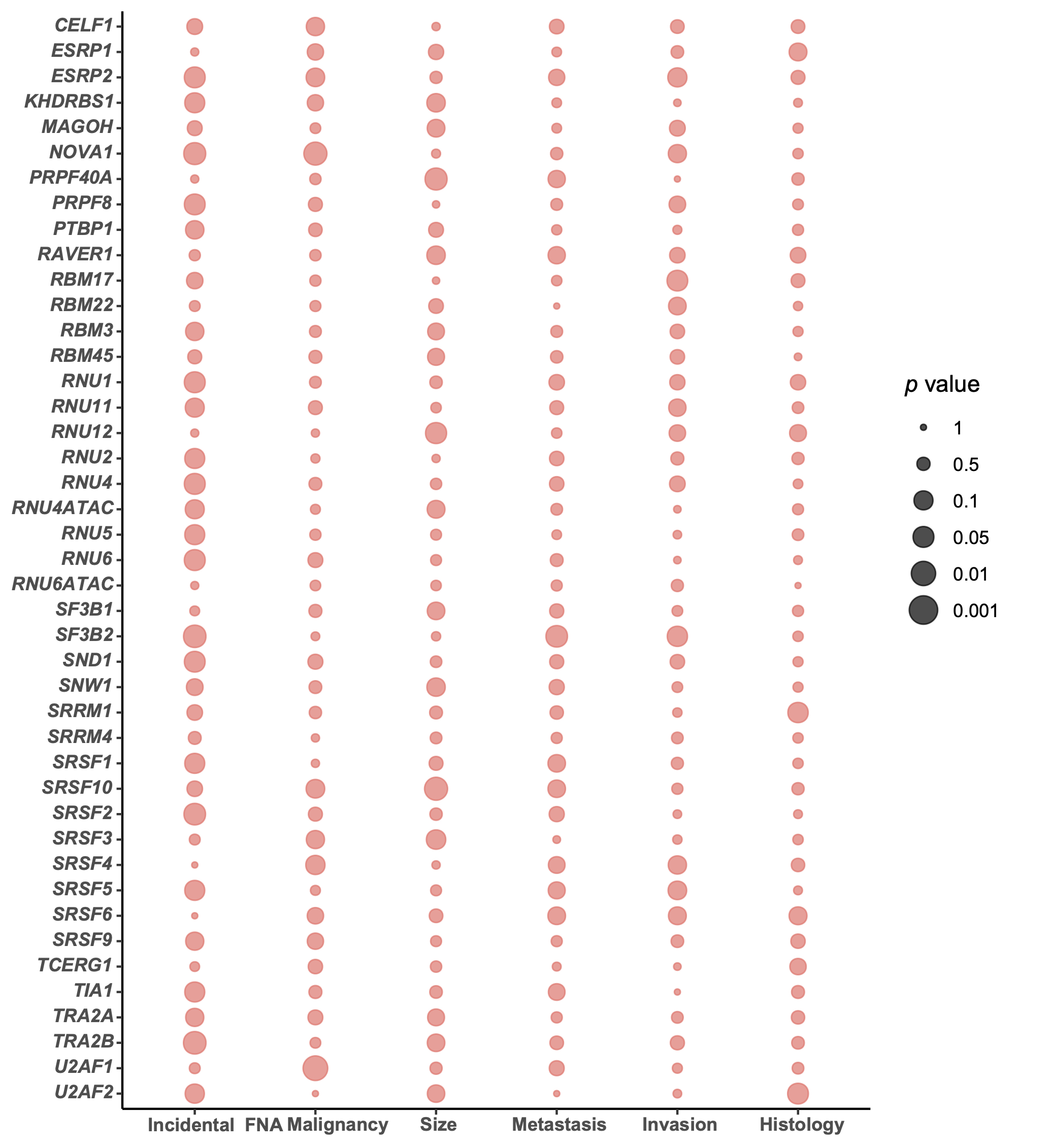

Supplement: Supplementary file 2 — Additional file 2: Figure S2. Association of the expression levels of components of the splicing machinery with different relevant clinical parameters in the Discovery cohort. The size of the circles refers to the p value of the clinical association [file 12967_2023_4754_MOESM2_ESM.jpg]

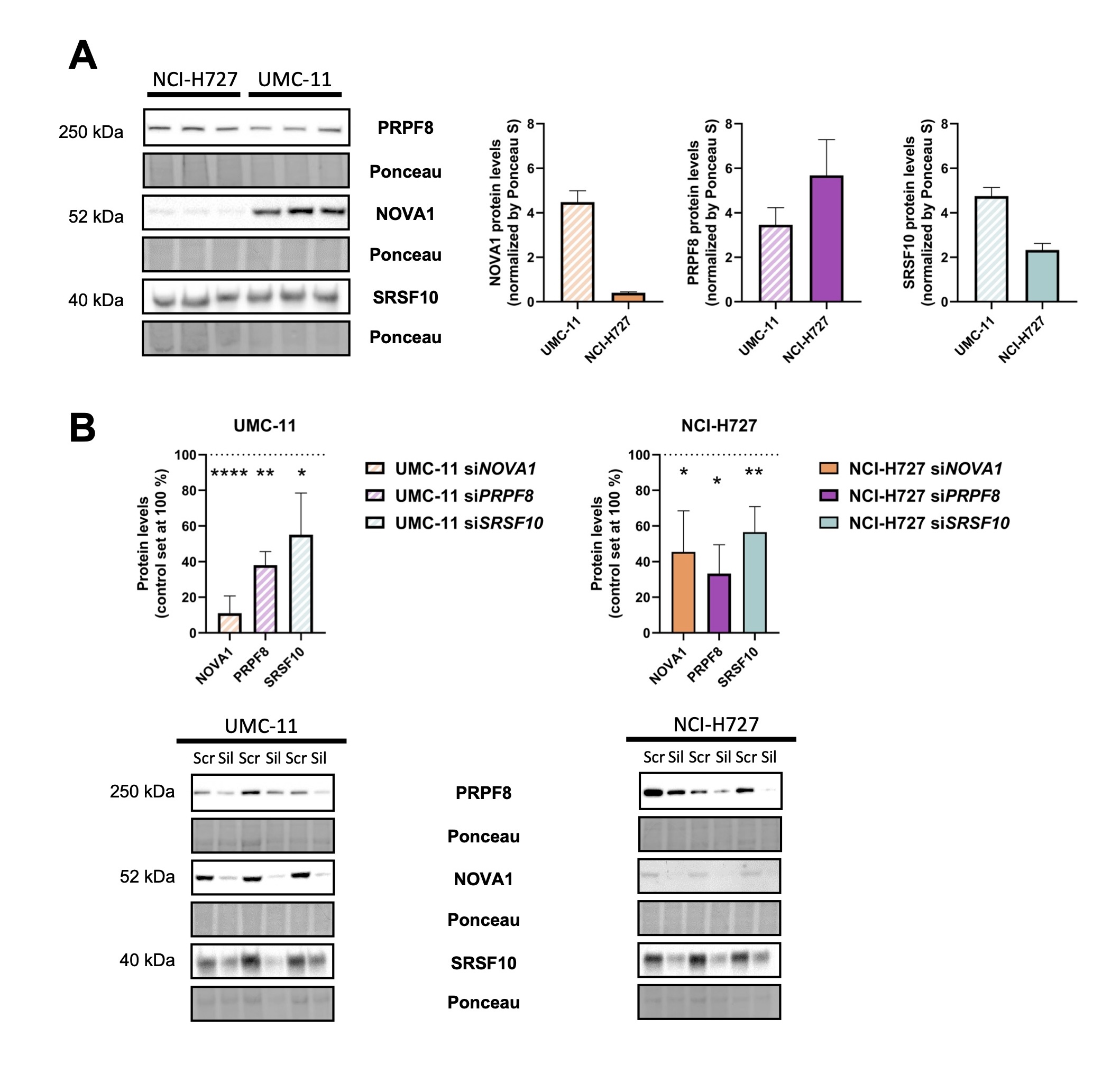

Supplement: Supplementary file 3 — Additional file 3: Figure S3A. Protein levels of NOVA1, PRPF8 and SRSF10 in model cell lines under basal conditions (n = 3) as assessed by Western Blot analysis. B. Protein levels of NOVA1, PRPF8 and SRSF10 in model cell lines after respective gene silencing using specific siRNA (n = 3). Data were normalized with Ponceau and represented as percentage compared to Scramble (set at 100%). Data represents mean ± SD. Asterisks indicate values that significantly differences between groups (*, p < 0.05; **, p < 0.01; ****, p < 0.0001) [file 12967_2023_4754_MOESM3_ESM.jpg]
